# Relationship between annualized case volume and in-hospital motality in subarachnoid hemorrhage: A systematic review and meta-analysis

**DOI:** 10.1097/MD.0000000000027852

**Published:** 2021-12-03

**Authors:** Jian-Yi Huang, Hong-Yu Lin, Qing-Qing Wei, Xing-Hua Pan, Ning-Chao Liang, Wen Gao, Sheng-Liang Shi

**Affiliations:** a Department of Neurology, People's Hospital of Chongzuo city, Chongzuo, Guangxi Zhuang Autonomous Region, China; b Department of Neurology, People's Hospital of Liuzhou city, Liuzhou, Guangxi Zhuang Autonomous Region, China; c Department of Neurology, Second Affiliated Hospital of Guangxi Medical University, Nanning, Guangxi Zhuang Autonomous Region, China.

**Keywords:** case volume, hospital volume, in-hospital motality, meta-analysis, subarachnoid hemorrhage

## Abstract

Studies on the relationship between hospital annualized case volume and in-hospital mortality in patients with subarachnoid hemorrhage (SAH) have shown conflicting results. Therefore, we performed a meta-analysis to further examine this relationship.

The authors searched the PubMed and Embase databases from inception through July 2020 to identify studies that assessed the relationship between hospital annualized SAH case volume and in-hospital SAH mortality. Studies that reported in-hospital mortality in SAH patients and an adjusted odds ratio (OR) comparing mortality between low-volume and high-volume hospitals or provided core data to calculate an adjusted OR were eligible for inclusion. No language or human subject restrictions were imposed.

Five retrospective cohort studies with 46,186 patients were included for analysis. The pooled estimate revealed an inverse relationship between annualized case volume and in-hospital mortality (OR, 0.53; 95% confidence interval, 0.42–0.68, *P* < .0001). This relationship was consistent in almost all subgroup analyses and was robust in sensitivity analyses.

This meta-analysis confirms an inverse relationship between hospital annualized SAH case volume and in-hospital SAH mortality. Higher annualized case volume was associated with lower in-hospital mortality.

## 1 Introduction

Subarachnoid hemorrhage (SAH) accounts for 5% to 10% of all strokes in the United States.^[1]^ Although the incidence of SAH has not significantly changed over time, the total number of SAH hospital admissions and in-hospital SAH mortality have decreased. These decreases have been greater in large and extra-large hospitals than in smaller hospitals.^[2]^ Numerous studies have evaluated the relationship between hospital annual**ized** SAH case volume and SAH mortality;^[3–16]^ however, their results are conflicting. Hattori et al found no significant correlation between case volume and outcome for either ruptured or unruptured aneurysms.^[17]^ Johnston also concluded that outcomes were not significantly better in higher-volume institutions when adjusted for patient characteristics.^[18]^ In contrast, a 2014 systematic review found lower mortality in high-volume hospitals.^[19]^ However, this review analyzed crude data without adjusting for the confounders. In addition, numerous studies published after 2014 have also explored the relationship between SAH case volume and outcome. Therefore, we conducted an up-to-date systematic review and meta-analysis of this relationship.

## 2 Materials and methods

Ethical approval is not required because this article is a systematic review and meta-analysis.

### 2.1 Search strategy

A systematic literature search of the PubMed and Embase databases from database inception to July 2020 was conducted independently by 2 reviewers (HYL and JYH) to identify the relevant articles using the Meta-analysis of Observational Studies in Epidemiology checklist.^[20]^ No language or human subject restrictions were imposed. The search used key terms including “subarachnoid hemorrhage,” “volume,” “motality,” and their variants. Details of the search strategy are available in (Supplemental Digital Content Appendix S1) and (Supplemental Digital Content Appendix S2). We also manually searched the reference lists of all included studies and relevant reviews to identify other studies eligible for inclusion.

### 2.2 Study selection and eligibility criterias

Studies that reported in-hospital mortality in SAH patients and an adjusted odds ratio (OR) comparing mortality between low-volume and high-volume hospitals or provided core data to calculate an adjusted OR were eligible for study inclusion. After removal of duplicate studies, titles and abstracts were screened for relevance. The full text of potentially relevant studies was accessed and examined to determine eligibility.

### 2.3 Data extracion

Data extraction was performed by HYL and confirmed independently by 2 other authors (WG and QQW). The extracted information from each included study were as follows: first author, database, year of publication, country of study population, study subjects, study design, diagnostic criteria for SAH, main treatment modality, number of SAH cases, overall mortality rate, volume grouping (i.e., dichotomizations, tertiles, quartiles, quintiles, or other), volume categorization (i.e., category according to the various case volume cut-off values), multivariate adjusted risk estimates for each category, and covariates in the fully adjusted model. Data was outputted to a predetermined table.

### 2.4 Quality assessment

The methodological quality of each study was evaluated using the Newcastle-Ottawa Scale,^[21]^ which has been validated to assess the quality of nonrandomized studies in meta-analyses. This scale awards a maximum of 9 stars to each study: 4 stars for selection of participants and measurement of exposure, 2 stars for comparability, and 3 stars for assessment of outcomes and adequacy of follow-up. We defined scores of 0 to 3, 4, to 6, and 7 to 9 as low, moderate, and high quality of studies, respectively.

### 2.5 Statistical analysis

Statistical analyses were performed using STATA software version 12.0 (StataCorp LP, College Station, TX). Heterogeneity across studies was assessed using the *I*^2^ statistic, which is a quantitative measure of inconsistency across studies. Studies with an *I*^2^ index <25% were considered to have low heterogeneity, those with an *I*^2^ index 25% to 50% were considered to have moderate heterogeneity, and those with an *I*^2^ index >50% were considered to have high heterogeneity. A random-effects model was applied to pool multivariate ORs and their corresponding 95% confidence interval (CI) between extreme levels of annualized case volume (highest vs lowest) if there was high heterogeneity between studies. Otherwise, a fixed-effects model was used. Statistical tests for funnel plot asymmetry were not conducted given the limited specificity and power of these tests when fewer than 10 studies are included. Subgroup analyses were performed to explore possible sources of heterogeneity among studies according to: geographical region of study (Asian vs other continent), treatment modality (surgical clipping or endovascular treatment vs craniotocmy or trephination surgery vs unclear treatment modality), endpoint (14-day case-fatality rates vs 30-day mortality vs in-hospital mortality), annual**ized** case volume grouping (dichotomizations vs tertiles vs quartiles), and sample size (>10,000 vs <10,000). Furthermore, sensitivity analyses were performed to explore potential sources of heterogeneity and result robustness by omitting 1 study in each turn. Two-sided *P* < .05 was considered significant.

## 3 Results

### 3.1 Literature search

**Figure 1:**
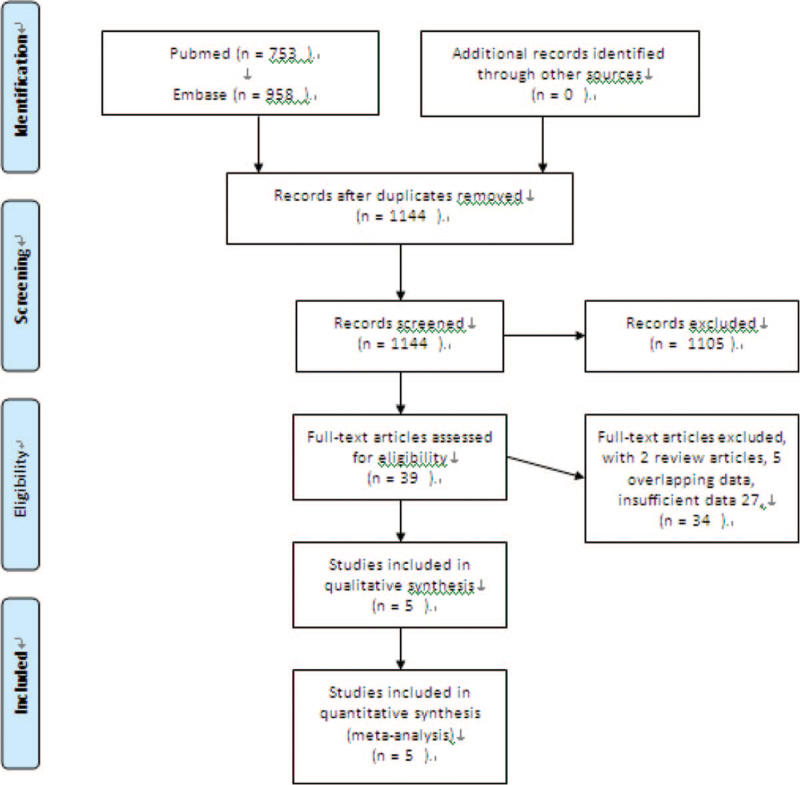
Flow diagram of the study selection process.

We identified 1144 articles in the initial search. After excluding duplicates and screening the titles and abstracts, 34 studies underwent full-text review. Among these, 5 had duplicated data,^[10,11,13,22,24]^ and 2 were reviews.^[19,23]^ Twenty seven were excluded because of insufficient data; among these, 3 only reported long-term mortality,^[25,26,27]^ 1 reported impact of teaching hospital status on mortality (not impact of hospitals volume),^[28]^ and 2 explored the transfer-outcome relationship.^[29,30]^ Finally, 5 studies were included in the quantitative meta-analysis.^[3,4,5,7,8]^ The study selection process is shown in Figure 1.

### 3.2 Study characteristics

The characteristics of the 5 included studies are shown in Table 1. All were retrospective cohort studies published between 2002 and 2019. The number of participants in the studies ranged from 355 to 18,944. Three studies came from Asia and 2 from Europe and the United States. Two did not report main therapeutic methods.^[7,8]^ In total, the 5 studies enrolled 46,186 patients. Crude in-hospital mortality ranged from 7.0% to 40%. Quality assessment of the included studies is shown in Table 2. The Newcastle-Ottawa Scale score was 5 for 1 study and 7 for the remaining 4, suggesting that all the studies were of moderate or high quality.

**Table 1:** Characteristics of the included studies.

Study	Database	Country	Study Design	Diagnostic criteria	Treatment Modality	Only Surgical patients	Endpoints	Number of Participants	In-hospital Mortality %	Volume Grouping	Volume Category Cases/Year	Number of each volume group	Deaths of each volume group	Multivariate OR (95%CI)	Covariates in Fully Adjusted Model
Bardach et al 2002	OSHPD hospital discharge database(January 1990 to December 1999)	US	Retrospective cohort	ICD 9 codes 430,excluding traumatic SAH and arteriovenous malformation	Surgical clipping or endovascular treatment	No	In-hospital mortality	12 804	40	Quartiles	<8	3154	1530	Ref	Age, sex, ethnicity, year of treatment, payment source, and admission acuity
											NR	3322	1369	0.78 (0.70-0.88)	
											NR	3207	1251	0.75 (0.64–0.87)	
											>19	3121	1008	0.58 (0.49–0.68)	
Lindgren et al 2019	Dr Foster Stroke GOAL database (2007–2014)	Europe,US and Australia	Retrospective cohort	ICD 9 codes 430 and ICD 10 codes I60.0–9	Surgical clipping or endovascular treatment	Yes	14-day case-fatality rates	8525	7.46	Tertiles	<41	2363	246	Ref	Age, sex, aneurysm treatment modality, and severity and comorbidity markers
											41-70	3563	250	0.63 (0.47–0.85)	
											>70	2599	140	0.50 (0.33–0.74)	
Lin et al 2014	National Health Insurance Research Database of Taiwan (2000–2009)	Taiwan	Retrospective cohort	ICD 9 codes 430, excluding 800.0–801.9, 803.0–804.9, 850.0–854.1, and 873.0–873.9	NR	No	Mortality within 30 days of admission	355	7.0	Dichotomizations	≤30	NR	NR	Ref	Sex, surgeon volume, hospital level (medical center versus nonmedical center hospital),and CCI
											>30	NR	NR	0.277 (0.091–0.842)	
Lee et al 2018	Health Insurance Review and Assessment Service (2009–2013)	Korea	Retrospective cohort	ICD-10 codes I60, excluding traumatic SAH	Craniotomy or trephination surgery	Yes	Mortality within 30 days of admission	18944	12.9	Tertiles	NR	5383	840	Ref	Age, sex, hemorrhage site, social security system, intensive care unit admission, hypertension,and CCI
											NR	6327	800	0.78 (0.70–0.87)	
											NR	7234	806	0.68 (0.61–0.76)	
Tsugawa et al 2013	DPC inpatient database (July 2010 to December 2010)	Japan	Retrospective cohort	ICD-10 codes I60	NR	No	In-hospital mortality	5558	NR	Tertiles	10–50	NR	NR	4.42 (2.21–8.83)	Age, sex, modified Rankin Scale, use of mechanical ventilation, comorbidities (renal failure, heart failure, malignant neoplasm), hospital ownership, and nurse-to-bed ratio
											51–100	NR	NR	1.54 (1.13–2.08)	
											>100	NR	NR	Ref	

**Table 2:** Methodological quality assessment of included studies by Newcastle-Ottawa Scales.

	Selection					Outcome			
Study	Exposed cohort	Nonexposed cohort	Ascertainment of exposure	Outcome of interest	Comparability	Assessment of outcome	Length of follow-up	Adequacy of follow-up	Total score
Bardach et al 2002	^∗^	^∗^	^∗^	^∗^	^∗∗^	^∗^	—	—	7
Lindgren et al 2019	^∗^	^∗^	^∗^	^∗^	^∗∗^	^∗^	—	—	7
Lin et al 2014	^∗^	^∗^	^∗^	^∗^	—	^∗^	—	—	5
Lee et al 2018	^∗^	^∗^	^∗^	^∗^	^∗∗^	^∗^	—	—	7
Tsugawa et al 2013	^∗^	^∗^	^∗^	^∗^	^∗∗^	^∗^	—	—	7

### 3.3 Relationship between case volume and in-hospital mortality

**Figure 2:**
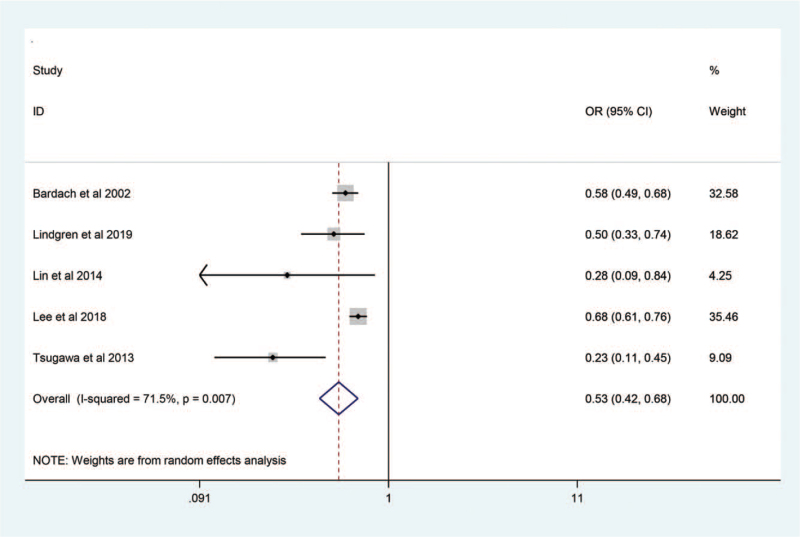
Forest plot of the relationship between annualized casevolume and in-hospital mortality among patients with subarachnoid hemorrhage.

High hospital case volume was significantly associated with reduced in-hospital mortality (OR 0.53; 95% CI, 0.42–0.68; *P* = .000; Fig. 2). However, study heterogeneity was significant (*I*^2^ = 71.5%; *P* = .007).

### 3.4 Subgroup analysis, sensitivity analyses, and publication bias

**Figure 3:**
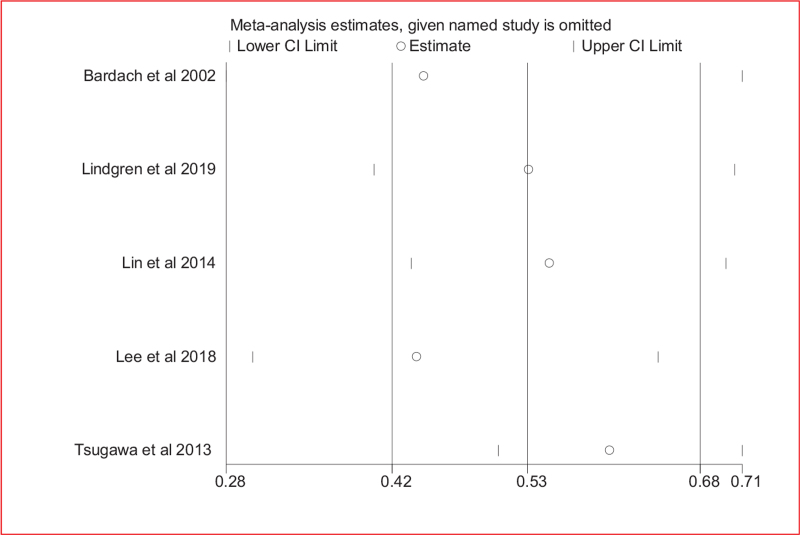
Sensitivity analyses for affirming the relationship between annualized casevolume and in-hospital mortality among patients with subarachnoid hemorrhage.

Table 3 shows the heterogeneity subgroup analyses according to geographical region, treatment modality, endpoint, annualized case volume grouping, sample size, and proportion of surgical patients. The relationship between annualized case volume and mortality was consistent in almost all subgroups. Exclusion of any single study from the meta-analysis did not significantly alter the magnitude or direction of the summary effect (Fig. 3).

**Table 3:** Subgroup analyses of relationship between annualized case volume and in-hospital mortality in subarachnoid hemorrhage.

Subgroup			Test of relationship		Test of heterogeneity	
		No. patients	OR (95%CI)	*P* value	*I*^2^, %	*P* value
Region	Asian^[4,7,8]^	24,857	0.38 (0.16–0.89)	.026	82.2	.004
	Other region^[3,5]^	21,329	0.57 (0.49–0.66)	.000	0.0	.504
Treatments	Surgical clipping or endovascular treatment^[3,5]^	21,329	0.57 (0.49–0.66)	.000	0.0	.504
	Craniotomy or trephination surgery^[4]^	18,944	0.68 (0.61–0.76)	.000	NA	NA
	NR^[7,8]^	5913	0.24 (0.13–0.44)	.000	0.0	.782
Endpoints	In-hospital mortality^[3,8]^	18,362	0.39 (0.16–0.96)	.04	84.1	.012
	Mortality within 30 d of admission^[4,7]^	19,299	0.52 (0.23–1.16)	.11	59.7	.115
	14-day case-fatality rates^[5]^	8525	0.50 (0.33–0.74)	.001	NA	NA
Volume grouping	Dichotomizations^[7]^	355	0.277 (0.091–0.842)	.024	NA	NA
	Tertiles^[4,5,8]^	33,027	0.48 (0.29–0.78)	.004	81.2	.005
	Quartiles^[3]^	12,804	0.58 (0.49–0.68)	.000	NA	NA
Sample size	< 10,000^[5,7,8]^	31,748	0.35 (0.20–0.61)	.000	49.5	.138
	> 10,000^[3,4]^	14,438	0.64 (0.54–0.74)	.000	59.9	.114
Surgical patiets	All^[4,5]^	27,469	0.62 (0.47–0.82)	.001	51.8	.150
	Part^[3,7,8]^	18,717	0.37 (0.18–0.75)	.006	74.2	.021

## 4 Discussion

The results of our meta-analysis confirm an inverse relationship between hospital annualized SAH case volume and in-hospital SAH mortality: hospitals with higher annualized case volume had lower in-hospital mortality. This relationship was robust and consistent in subgroup analyses. A previous review published in 2014 that compared SAH outcomes between high-volume and low-volume centers also showed lower mortality in high-volume centers (OR 0.77; 95% CI, 0.60–0.97; *P* = .029).^[19]^ However, no attempt was made to adjust for potential confounders such as age, sex, comorbidities, SAH severity or hospital status, which reduces the robustness of their results. In addition, SAH outcomes based on treatment method (endovascular vs open surgery) were emphasized in this previous review. In our systematic review, we used an adjusted OR to explore the volume-outcome relationship and demonstrated that higher SAH case volume is associated with lower in-hospital mortality. Moreover, we did not limited the treatment modalities for SAH patients because these patients could be received 1 or more treatments such as clipping, endovascular coiling, trephination, craniotomy and bone flap decompression and could be limited to these aggressive treatments. In the subgroup analyses, we analyzed the pooled ORs separately by dividing the studies into those that only included surgical patients (*P* = .001) and those that included patients who received any treatment (*P* = .006). The results were consistent in each subgroup.

Luft et al ^[31]^ were the first to report that the number of procedures performed in a hospital was inversely related to procedure-related mortality. The volume-outcome relationship is probably caused by the “practice-makes-perfect” and selective-referral pattern theories. The former states that increased frequency of encounters allows higher case volume centers to develop more experience and streamline processes to improve quality of care. The latter implies that patients disproportionately seek care at, and physicians refer to, hospitals known for high quality of care. Therefore, high volume and high quality are interrelated. SAH patients are critically ill and have a 15 times higher risk of a second hemorrhagic event than the general population.^[32]^ A second hemorrhagic event is often fatal. These patients usually cannot choose the hospital where they are treated because of the acute presentation and severe neurologic effects of their disease. Nuño et al^[15]^ reported that 32.7% of aneurysmal SAH patients are treated after interhospital transfer and that transfer and direct-admit patients have comparable mortality and complications. Therefore, there is reason to believe that “practice-makes-perfect” could play a role in improving quality of care.

Our findings suggest that centralization of care might benefit SAH patients. Furthermore, transferring SAH patients who arrive at low-volume hospitals to high-volume hospitals is probably cost-effective.^[33]^ However, this could overburden clinical resources in the centralized centers. Therefore, the trade-offs between the risks and benefits associated with centralization must be weighed.

This meta-analysis has several limitations. First, the included studies were all retrospective and study heterogeneity was considerable. To reduce bias as much as possible, we used a random-effects model to pool multivariate estimates and performed subgroup and sensitivity analyses to explore potential sources of heterogeneity and robustness. Second, confounding may have affected our results since the data was based on hospital coding and our ability to control for confounders was limited. Finally, we did not explore publication bias since only 5 studies were included; current guidelines do not recommend testing for funnel plot asymmetry in analyses of fewer than 10 studies.^[34]^

## 5 Conclusions

In conclusion, this meta-analysis confirms an inverse relationship between hospital annualized SAH case volume and in-hospital SAH mortality. Higher case volume was associated with lower in-hospital mortality. Future studies that examine the SAH case volume–mortality relationship are warranted. These studies should include adjustments for annualized case volume and treatment modality. Standardized definitions of high and low case volumes are needed.

## Author contributions

**Conceptualization:** Hong-Yu Lin, Sheng-Liang Shi.

**Data curation:** Hong-Yu Lin.

**Formal analysis:** Jian-Yi Huang, Wen Gao.

**Investigation:** Qing-Qing Wei, Xing-Hua Pan, Ning-Chao Liang.

**Methodology:** Jian-Yi Huang.

**Project administration:** Jian-Yi Huang, Qing-Qing Wei, Wen Gao, Sheng-Liang Shi.

**Software:** Hong-Yu Lin.

**Supervision:** Xing-Hua Pan, Ning-Chao Liang.

**Validation:** Jian-Yi Huang, Qing-Qing Wei, Xing-Hua Pan, Ning-Chao Liang, Wen Gao.

**Visualization:** Qing-Qing Wei, Xing-Hua Pan, Ning-Chao Liang.

**Writing – original draft:** Jian-Yi Huang.

**Writing – review & editing:** Hong-Yu Lin, Sheng-Liang Shi.

## Supplementary Material

Supplemental Digital Content
