# Supplementary material for: Relationship between annualized case volume and in-hospital motality in subarachnoid hemorrhage: A systematic review and meta-analysis
Source: Medicine (Baltimore). 2021 Dec 3;100(48):e27852. doi: 10.1097/MD.0000000000027852 (PMC9191364; doi:10.1097/MD.0000000000027852)
Supplement: Supplemental Digital Content [file medi-100-e27852-s001.docx]

Supplemental Digital Content (Appendix 1). Detailed search strategy in Embase

Supplemental Digital Content (Appendix 2). Detailed search strategy in Pubmed
